# Analysis of patients with differing short-term rates of improvement and long-term rates of decline in range of motion and after anatomic and reverse total shoulder arthroplasty

**DOI:** 10.1016/j.jseint.2025.04.018

**Published:** 2025-05-14

**Authors:** Christopher P. Roche, Josie Elwell, Richard Jones, Howard Routman, Ryan Simovitch, Pierre-Henri Flurin, Thomas W. Wright, Joseph D. Zuckerman

**Affiliations:** a Exactech, Gainesville, FL, USA; b Southeastern Sports Medicine, Asheville, NC, USA; c Atlantis Orthopedics, Palm Beach Gardens, FL, USA; d Hospital for Special Surgery, West Palm Beach, FL, USA; e Bordeaux-Merignac Sport Clinic, Merignac, France; f Department of Orthopaedic Surgery, University of Florida, Gainesville, FL, USA; g Department of Orthopedic Surgery at NYU Langone Orthopedic Hospital, New York, NY, USA

**Keywords:** Rate of improvement, Rate of decline, Range of motion, Clinical outcomes, Anatomic total shoulder arthroplasty, Reverse total shoulder arthroplasty

## Abstract

**Background:**

Patients with anatomic (aTSA) and reverse total shoulder arthroplasty (rTSA) can have different short-term rate of improvement (ROI) and different long-term rate of decline (ROD) in range of motion (ROM). This study aims to quantify and compare these rates and identify risk factors associated with a slow ROI and a fast ROD after both aTSA and rTSA.

**Methods:**

This 8-year minimum longitudinal outcome study compares active ROM in 1272 primary aTSA (n = 688) and rTSA (n = 584) patients across 8357 visits and identified patient cohorts with a slow, average, and fast ROI from 0-2 years after surgery and a slow, average, and fast ROD 8 years after surgery relative to peak improvement achieved 2-3 years after surgery. A multivariate regression analysis was performed to identify patient, implant/operative, or postoperative risk factors associated with a slow ROI and fast ROD after both aTSA and rTSA.

**Results:**

The results of this 1272 patient long-term clinical outcome study demonstrates that aTSA and rTSA patients with a slow ROI were associated with high preoperative ROM and patients with a fast ROI were associated with low preoperative ROM. aTSA and rTSA patients with high preoperative ROM experienced declines in ROM during the first 3 months, but later recovered at a similar rate and achieved similar peak improvements. aTSA patients with a slow ROI had significantly higher preoperative abduction, internal rotation score, and external rotation, whereas rTSA patients with a slow ROI were significantly more likely to have diabetes, injections, and significantly higher preoperative abduction and internal rotation score. aTSA patients with a fast ROD were significantly more likely to have heart disease and glenoid radiolucent lines, whereas rTSA patients with a fast ROD were significantly more likely to have comorbidities and experience revision surgery.

**Discussion:**

The rate of improvement in ROM during the short-term recovery period after aTSA and rTSA is highly dependent on preoperative ROM, whereas the rate of decline in ROM at long-term follow-up is generally impacted by systemic health issues (ie, heart disease and more comorbidities), compromised implant fixation (ie, radiolucent lines after aTSA), and the onset of revision surgery. These findings may be beneficial for patient counseling and expectation management, especially to encourage patients who may have experienced a decline in ROM during the first 3 months after surgery.

Patients with a variety of degenerative conditions of the shoulder reliably achieve improvement in active range of motion (ROM) following anatomic (aTSA) and reverse total shoulder arthroplasty (rTSA).^5^^,^^6^^,^^22^^,^^25^^,^^30^^,^^31^^,^^41^ aTSA and rTSA patients can experience improvements in ROM throughout the first few years after surgery, with peak improvement achieved 2-3 years postoperatively.^9^^,^^13^^,^^21^^,^^40^^,^^38^ However, the rate of improvement (ROI) during this recovery period varies depending on length of follow-up and prosthesis type.^15^^,^^20^^,^^39^ Moreover, some aTSA and rTSA patients may achieve faster ROI in ROM than others. Shoulder arthroplasty patients can also experience a decline in ROM with long-term follow-up^1^^,^^10^^,^^11^^,^^17^^,^^27^^,^^35^^,^^36^; some patients may experience a faster rate of decline (ROD) in ROM than others. The reasons for this decline are multifactorial, differ between prosthesis type/design, and are likely influenced by patient age,^33^ comorbidities, activity level, soft tissue integrity, prosthesis-boney fixation, and prosthesis durability.^2^^,^^3^^,^^6^^,^^7^^,^^8^^,^^14^^,^^16^^,^^17^^,^^18^^,^^19^^,^^20^^,^^21^^,^^22^^,^^23^^,^^23^^,^^24^^,^^25^^,^^26^^,^^27^^,^^34^^,^^35^^,^^36^^,^^37^^,^^42^

It is unknown why some aTSA and rTSA patients achieve a slower ROI in ROM during the recovery period and also unknown why some achieve a faster ROD in ROM at the long-term follow-up. Therefore, the aims of this study are (1) to quantify and compare the ROI of ROM during the 2-year recovery period and quantify and compare the ROD in ROM from short-term to long-term follow-up after aTSA and rTSA and (2) to stratify aTSA and rTSA patients into cohorts who experience a slow, average, and fast ROI in ROM during the 2-year recovery period and a slow, average, and fast ROD in ROM at long-term follow-up and identify any patient or implant/operative parameters associated with a slow ROI or a fast ROD after aTSA and rTSA.

## Methods

A retrospective review of a multicenter clinical outcomes database of shoulder arthroplasty patients who received a single platform shoulder prosthesis (Equinoxe; Exactech, Inc, Gainesville, FL, USA) was performed for all patients with 8 years minimum follow-up. Every patient provided consent and all data were collected using standardized forms according to an institutional review board–approved protocol. Clinical data from 1495 shoulder arthroplasty patients were considered. Patients who did not have follow-up of at least 8 years were excluded. To ensure a homogenous dataset of primary aTSA and rTSA patients, patients with a diagnosis of revision arthroplasty (n = 129), diagnosis of proximal humeral fracture (n = 55), as well as patients with hemiarthroplasty (n = 38) and resurfacing humeral heads (n = 1) were excluded, leaving 1272 patients available for inclusion. These 1272 patients (714 F, 553 M, 5 Unk) consisted of 688 primary aTSA patients (335 F, 349 M, 4 Unk) and 584 primary rTSA (379 F, 204 M, 1 Unk), with a total of 8357 visits (4535 aTSA, 3822 rTSA). The mean age of the aTSA patient cohort at the time of surgery was 65.0 ± 8.2 years (range: 31-85) and the mean age of the rTSA patient cohort at the time of surgery was 70.7 ± 6.8 years (range: 38-87). The average follow-up at the latest clinic visit was 113.6 ± 25.0 months (aTSA: 118.1 ± 28.9, rTSA: 108.4 ± 17.9).

Patients were evaluated preoperatively and at multiple postoperative timepoints using the American Shoulder and Elbow Surgeons, Constant, and Shoulder Arthroplasty Smart^32^ scoring metrics. Pain was quantified using the 11-point visual analog scale and shoulder function was quantified using the 11-point global shoulder function score. Active abduction, forward elevation, and internal rotation (IR), and external rotation were also measured. IR was measured by the IR score.^5^ Radiographic outcomes and revision rates were also analyzed.

The ROI for each ROM measure was calculated for each patient, for each recovery period visit (defined as 0-24 months), by dividing each patient's preoperative to postoperative ROM improvement at each visit, by that patient's follow-up duration for a given visit. Average ROI for each ROM measure for each postoperative interval was calculated separately for aTSA and rTSA patients. aTSA and rTSA patients were classified into the “fast ROI” group if >two-third of their ROI measures exceeded the average ROI associated with each prosthesis cohort and classified into the “slow ROI” group if <one-third of their ROI measures exceeded the average ROI associated with each prosthesis cohort. Finally, aTSA and rTSA patients were classified into the “average ROI” group if between one-third and two-third of the ROI measures exceeded the average ROI associated with each prosthesis cohort.

Peak improvement for each ROM measure was identified for each patient by selecting the highest value of each ROM measure across each visit during the 2-3 year postoperative interval. The ROD for each ROM measure was calculated for each patient, for each long-term follow-up visit (defined as 96+ months) by subtracting each patient's peak ROM from their ROM value at each long-term visit timepoint and then dividing that ROM difference by that patient's follow-up duration for a given long-term visit. Average ROD for each ROM measure for each postoperative interval was calculated separately for aTSA and rTSA patients. aTSA and rTSA patients were classified into the “fast ROD” group if >two-third of their ROD measures exceeded the average ROD associated with each prosthesis cohort and classified into the “slow ROD” group if <one-third of their ROD measures exceeded the average ROD associated with each prosthesis cohort. Finally, aTSA and rTSA patients were classified into the “average ROD” group if between one-third and two-third of the ROD measures exceeded the average ROD associated with each prosthesis cohort.

A logistic multivariate regression was performed to identify the factors (patient demographics/comorbidities, implant/operative parameters, and postoperative/radiographic parameters) associated with (1) patients with a fast/average ROI in ROM vs. patients with a slow ROI in ROM during the recovery period and (2) patients with a slow/average ROD in ROM vs. patients with a fast ROD in ROM at long-term follow-up. Specifically, a univariate analysis was performed using a 2-tailed, unpaired t-test for continuous variables and a Fisher's Exact test for discrete variables. Next, a multivariate logistic regression analysis was performed on the parameters identified as being significant from the univariate analysis to determine the significance and odds ratio of the variables associated with a slow ROI during the recovery period and a fast ROD at long-term follow-up. The multivariate analysis evaluated and adjusted the effect of each individual parameter, holding all the other variables constant. Odds ratios were calculated with a 95% confidence interval, with the significance level set at 0.05.

## Results

**Figure 1:**
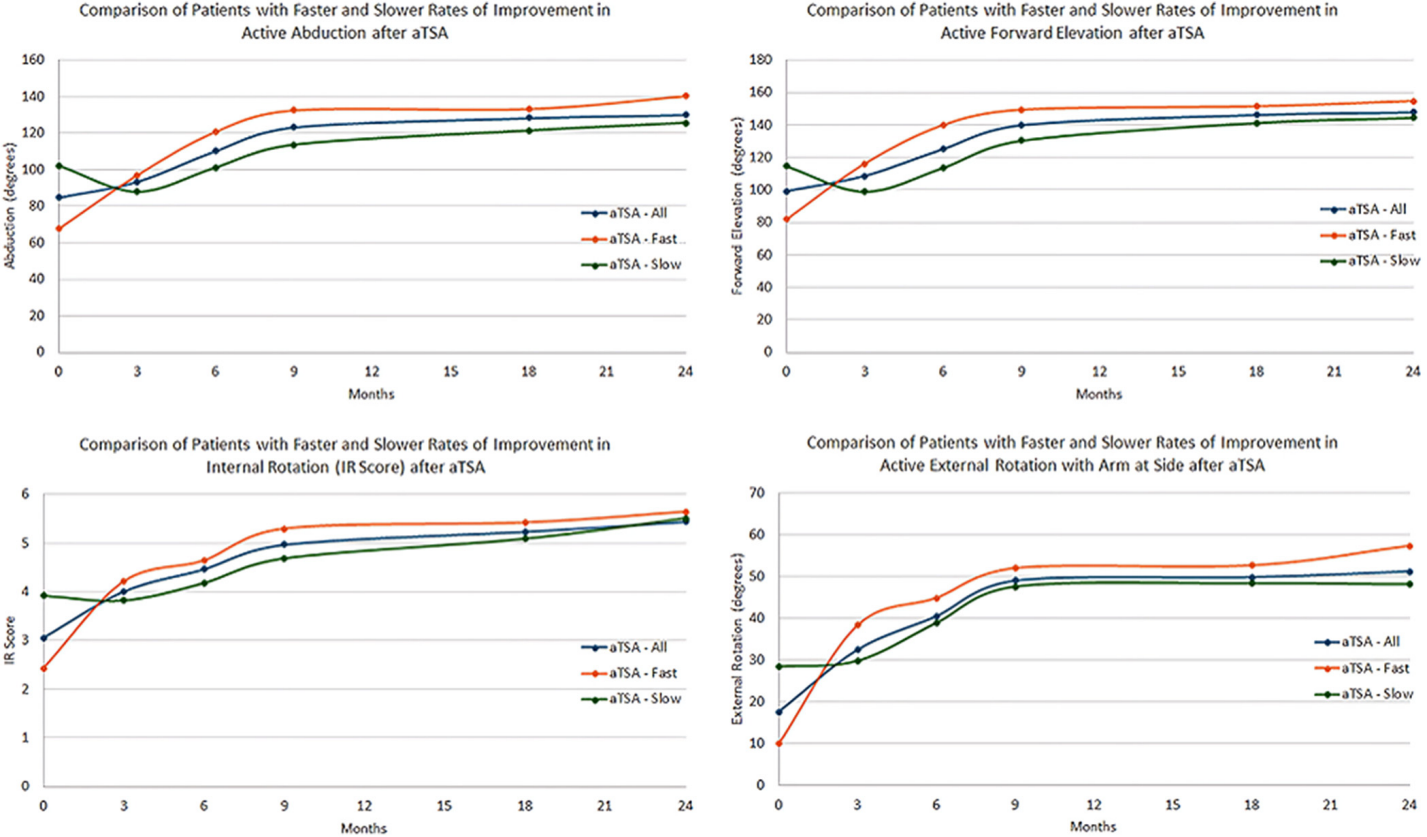
Comparison of active ROM in abduction (*top left*), forward elevation (*top right*), IR score (*bottom left*), and external rotation with arm at side (*bottom right*) for aTSA patients for patients with a fast ROI vs. slow ROI in ROM at short-term (0-2 years) follow-up. *aTSA*, anatomic total shoulder arthroplasty; *IR*, internal rotation; *ROI*, rate of improvement; *ROM*, range of motion.

**Figure 2:**
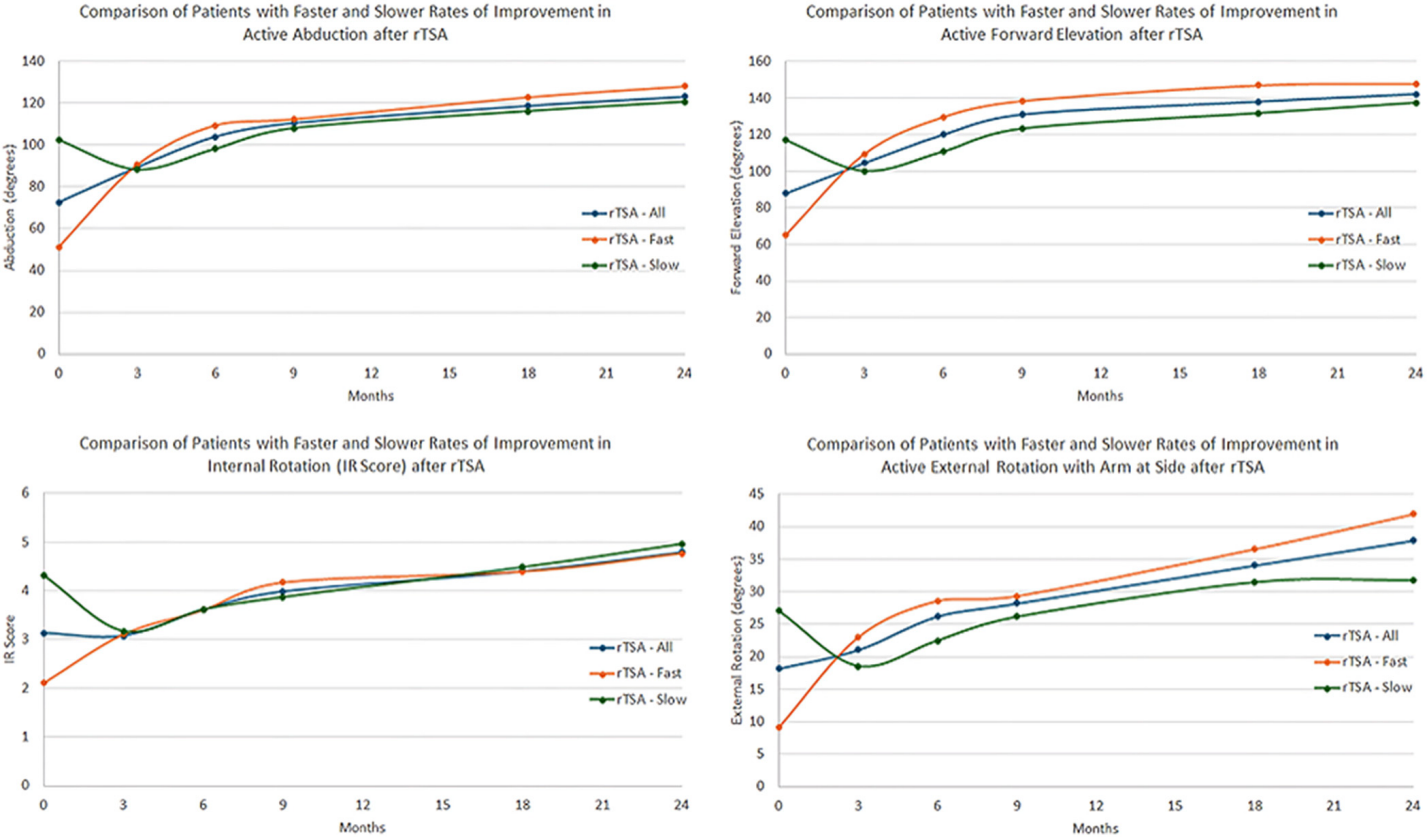
Comparison of active ROM in abduction (*top left*), forward elevation (*top right*), IR score (*bottom left*), and external rotation with arm at side (*bottom right*) for rTSA patients for patients with a fast ROI vs. slow ROI in ROM at short-term (0-2 years) follow-up. *rTSA*, reverse total shoulder arthroplasty; *IR*, internal rotation; *ROI*, rate of improvement; *ROM*, range of motion.

aTSA and rTSA patients with a fast ROI in ROM had significantly worse/lower values preoperatively for every clinical measure as compared to aTSA and rTSA patients with a slow ROI, except for global shoulder function score with rTSA patients (aTSA: Table I and Fig. 1, rTSA: Table II and Fig. 2). At the latest long-term follow-up for aTSA patients, no differences in any postoperative clinical outcome measures were observed between aTSA patients with a fast or slow ROI (Table III). At the latest follow-up for rTSA patients, only a few differences were observed between fast and slow ROI cohorts, where specifically, rTSA patients with a fast ROI had significantly higher active abduction (*P* = .0295), significantly less visual analog scale pain (*P* = .0368), and a significantly higher American Shoulder and Elbow Surgeons score (*P* = .0045), as compared to rTSA patients with a slow ROI (Table IV).

**Table I:** Comparison of preoperative outcomes for primary aTSA patients with slow and fast ROI in ROM from 0 to 2 years.

Follow-up duration	Active abduction	Active forward elevation	Active external rotation	IR score	VAS pain	Global shoulder function	ASES	Constant	Shoulder arthroplasty smart
Full aTSA cohort Preoperative	84.8 ± 29.7	99.2 ± 31.5	17.6 ± 20.1	3.1 ± 1.6	6.4 ± 2.0	4.0 ± 2.0	35.5 ± 15.6	38.0 ± 13.4	45.9 ± 10.4
Fast ROI aTSA Preoperative	67.6 ± 21.0	81.8 ± 25.4	9.9 ± 17.5	2.4 ± 1.4	6.7 ± 2.0	3.8 ± 2.0	31.8 ± 14.1	31.8 ± 10.8	41.0 ± 9.0
Slow ROI aTSA Preoperative	102.2 ± 29.7	114.6 ± 30.3	28.4 ± 18.2	3.9 ± 1.5	5.9 ± 2.2	4.6 ± 2.1	40.4 ± 16.3	46.0 ± 13.8	53.1 ± 9.3
*P* value (fast ROI vs. slow ROI)	<.0001	<.0001	<.0001	<.0001	.0005	.0004	<.0001	<.0001	<.0001

*aTSA*, anatomic total shoulder arthroplasty; *ROI*, rate of improvement; *IR*, internal rotation; *ROM*, range of motion; *ASES*, American Shoulder and Elbow Surgeons; *VAS*, visual analog scale.

**Table II:** Comparison of preoperative outcomes for primary rTSA patients with slow and fast ROI in ROM from 0 to 2 years

Follow-up duration	Active abduction	Active forward elevation	Active external rotation	IR score	VAS pain	Global shoulder function	ASES	Constant	Shoulder arthroplasty smart
Full rTSA cohort Preoperative	72.6 ± 34.5	88.0 ± 38.9	18.2 ± 21.6	3.1 ± 1.9	6.0 ± 2.3	3.7 ± 2.0	36.3 ± 16.1	34.6 ± 14.0	45.5 ± 12.0
Fast ROI rTSA Preoperative	51.2 ± 22.3	65.1 ± 27.5	9.1 ± 18.2	2.1 ± 1.7	6.4 ± 2.3	3.5 ± 2.1	32.5 ± 14.3	26.6 ± 10.3	38.2 ± 9.3
Slow ROI rTSA Preoperative	102.5 ± 32.5	117.0 ± 36.0	27.1 ± 21.5	4.3 ± 1.8	5.7 ± 2.4	4.0 ± 2.2	41.0 ± 17.8	44.2 ± 14.5	53.8 ± 12.2
*P* value (fast ROI vs. slow ROI)	<.0001	<.0001	<.0001	<.0001	.0081	.0574	<.0001	<.0001	<.0001

*rTSA*, reverse total shoulder arthroplasty; *ROI*, rate of improvement; *IR*, internal rotation; *ROM*, range of motion; *ASES*, American Shoulder and Elbow Surgeons; *VAS*, visual analog scale.

**Table III:** Comparison of latest follow-up outcomes for primary aTSA patients with slow and fast ROI in ROM from 0 to 2 years.

Follow-up duration	Active abduction (post/improve)	Active forward elevation (post/improve)	Active external rotation (post/improve)	IR score (post/improve)	VAS pain (post/improve)	Global shoulder function (post/improve)	ASES (post/improve)	Constant (post/improve)	Shoulder arthroplasty smart (post/improve)
Full aTSA cohort Latest follow-up of 118.1 mo	115.8 ± 33.6/30.4 ± 37.4	138.2 ± 33.3/37.6 ± 38.0	43.8 ± 19.4/27.2 ± 23.4	4.5 ± 1.6/1.6 ± 1.9	2.0 ± 2.7/4.6 ± 3.2	7.8 ± 2.3/3.9 ± 2.8	77.8 ± 23.5/43.5 ± 26.4	63.1 ± 16.8/24.1 ± 18.1	72.9 ± 15.6/27.8 ± 16.1
Fast ROI aTSA Latest follow-up of 119.4 mo	114.7 ± 32.1/48.3 ± 33.0	136.1 ± 33.8/54.6 ± 36.3	46.2 ± 19.3/37.7 ± 21.4	4.5 ± 1.5/2.0 ± 1.7	1.7 ± 2.7/5.0 ± 3.4	8.0 ± 2.3/4.1 ± 3.0	79.7 ± 23.8/47.1 ± 27.6	62.0 ± 18.2/28.5 ± 17.5	73.9 ± 15.5/32.4 ± 16.1
Slow ROI aTSA Latest follow-up of 116.108 mo	113.6 ± 35.5/13.5 ± 32.3	134.3 ± 37.9/20.0 ± 33.9	44.1 ± 18.9/16.0 ± 21.5	4.7 ± 1.5/0.8 ± 1.7	1.9 ± 2.6/4.3 ± 3.2	7.8 ± 2.3/3.4 ± 2.6	78.1 ± 23.7/40.3 ± 25.3	61.1 ± 18.2/17.3 ± 16.8	72.6 ± 17.1/21.2 ± 15.7
*P* value (fast ROI vs. slow ROI)	.8024/<.0001	.6815/<.0001	.3727/<.0001	.1937/<.0001	.6395/.0815	.3582/.0356	.5285/.0294	.7337/.0001	.5512/<.0001

*aTSA*, anatomic total shoulder arthroplasty; *ROI*, rate of improvement; *IR*, internal rotation; *ROM*, range of motion; *ASES*, American Shoulder and Elbow Surgeons; *VAS*, visual analog scale.

**Table IV:** Comparison of latest follow-up outcomes for primary rTSA patients with slow and fast ROI in ROM from 0 to 2 years

Follow-up duration	Active abduction (post/improve)	Active forward elevation (post/improve)	Active external rotation (post/improve)	IR score (post/improve)	VAS pain (post/improve)	Global shoulder function (post/improve)	ASES (post/improve)	Constant (post/improve)	Shoulder arthroplasty smart (post/improve)
Full rTSA cohort latest Follow-up of 108.4 mo	112.8 ± 29.2/36.4 ± 39.1	132.5 ± 28.3/39.6 ± 40.7	33.0 ± 18.8/14.6 ± 25.5	4.3 ± 1.8/1.0 ± 2.4	1.4 ± 2.3/4.6 ± 3.0	7.7 ± 2.1/4.0 ± 2.7	79.4 ± 20.1/42.9 ± 23.3	64.2 ± 16.4/27.2 ± 17.9	72.2 ± 14.0/24.8 ± 17.0
Fast ROI rTSA latest follow-up of 107.5 mo	116.5 ± 27.8/63.3 ± 29.8	136.8 ± 26.2/69.3 ± 31.2	34.6 ± 16.2/27.4 ± 22.4	4.6 ± 1.7/2.4 ± 2.1	1.2 ± 2.1/5.1 ± 2.9	7.9 ± 2.0/4.4 ± 2.7	82.3 ± 18.8/49.5 ± 22.2	66.6 ± 16.1/37.4 ± 14.3	74.1 ± 13.7/35.7 ± 14.2
Slow ROI rTSA latest follow-up of 106.5 mo	110.6 ± 29.3/10.7 ± 29.8	128.2 ± 30.5/11.2 ± 28.0	31.8 ± 19.4/6.5 ± 20.8	4.3 ± 1.8/-0.1 ± 2.0	1.7 ± 2.5/3.9 ± 3.2	7.5 ± 2.3/3.5 ± 2.9	76.0 ± 21.5/34.3 ± 22.9	63.1 ± 17.0/17.2 ± 15.8	70.3 ± 14.5/14.3 ± 14.4
*P* value (fast ROI vs. slow ROI)	.1867/<.0001	.0295/<.0001	.2603/<.0001	.2958/<.0001	.0368/.0003	.1202/.0046	.0045/<.0001	.1556/<.0001	.0560/<.0001

*rTSA*, reverse total shoulder arthroplasty; *ROI*, rate of improvement; *IR*, internal rotation; *ROM*, range of motion; *ASES*, American Shoulder and Elbow Surgeons; *VAS*, visual analog scale.

aTSA patients with a slow ROI had more ROM before surgery and on average experienced a decline in overhead ROM during the first 3 months (Fig. 1). rTSA patients in the slow ROI cohort had more ROM before surgery and experienced a decline in both overhead ROM and IR/external rotation during the first 3 months (Fig. 2). In contrast, both the fast ROI aTSA and rTSA cohorts had significantly less ROM before surgery and experienced substantial improvements in all ROM measures over the first 3 months. However, despite differences in preoperative ROM, the ROI (ie, the slope of the lines) between the fast, average, and slow ROI aTSA and rTSA cohorts were generally the same (ie, parallel) for each ROM measure between 3 and 24 months.

Differences between ROI cohorts were primarily driven by differences at the extremes of preoperative motion for both aTSA and rTSA patients. For example, aTSA and rTSA patients with <60° preoperative abduction achieved, on average, 38.9° and 43.7° improvement over the first 3 months after surgery, whereas patients with ≥120° preoperative abduction experienced, on average, 27.6° and 28.8° decline over the first 3 months, respectively. Similarly, aTSA and rTSA patients with <60° preoperative forward elevation achieved, on average, 43.0° and 54.6° improvement over the first 3 months after surgery, whereas patients with ≥120° preoperative forward elevation experienced, on average, 15.1° and 22.7° decline in abduction over the first 3 months, respectively. Moreover, aTSA and rTSA patients with preoperative abduction and forward elevation ≥120° generally did not experience ROM improvements until 6 to 12 months after surgery. Similar trends were observed for internal/external rotation.

Regarding peak improvement, aTSA patients with a fast ROI had significantly more active abduction (*P* = .0145), forward elevation (*P* = .0009), and external rotation (*P* = .0305) as compared to aTSA patients with a slow ROI, despite having significantly less preoperative ROM in each measure (Table V). Similarly, rTSA patients with a fast ROI had significantly more active forward elevation (*P* < .0001) and IR score (*P* = .0072) as compared to rTSA patients with a slow ROI, despite having significantly less preoperative ROM for each measure (Table V). Comparing peak improvement between prosthesis types demonstrates that aTSA patients had significantly more ROM for all measures (*P* < .0001) than rTSA patients.

**Table V:** Comparison of peak improvement in ROM outcomes for primary aTSA and rTSA patients with a slow and fast ROI in ROM at 2-3 Year follow-up.

2-3 yr follow-up duration	Active abduction - aTSA	Active forward elevation - aTSA	Active external rotation - aTSA	IR score - aTSA	Active abduction - rTSA	Active forward elevation - rTSA	Active external rotation - rTSA	IR score - rTSA
Full cohort peak improvement	131.9 ± 28.4	150.5 ± 22.8	53.3 ± 17.6	5.4 ± 1.2	121.1 ± 25.9	142.4 ± 21.3	35.7 ± 17.4	4.9 ± 1.6
Fast ROI peak improvement	137.2 ± 24.3	155.3 ± 18.4	57.1 ± 17.3	5.7 ± 1.0	125.3 ± 26.1	148.2 ± 17.0	38.1 ± 16.9	4.9 ± 1.5
Slow ROI peak improvement	129.2 ± 27.9	146.0 ± 25.3	53.1 ± 17.8	5.4 ± 1.3	119.7 ± 27.5	135.6 ± 22.5	32.0 ± 18.3	4.8 ± 1.7
*P* value (fast ROI vs. slow ROI)	.0145	.0009	.0305	.0693	.1043	<.0001	.8398	.0072

*aTSA*, anatomic total shoulder arthroplasty; *rTSA*, reverse total shoulder arthroplasty; *ROI*, rate of improvement; *IR*, internal rotation; *ROM*, range of motion.

**Figure 3:**
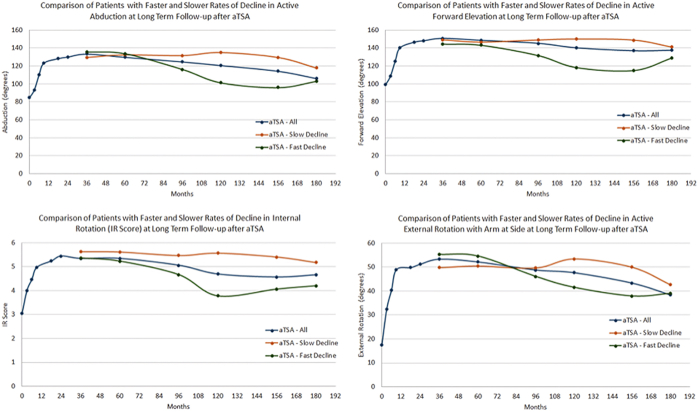
Comparison of active ROM in abduction (*top left*), forward elevation (*top right*), IR score (*bottom left*), and external rotation with arm at side (*bottom right*) for aTSA patients with a slow ROD (sustained outcomes) in ROM vs. fast ROD (not sustained outcomes) in ROM at long-term (>8 years) follow-up. *aTSA*, anatomic total shoulder arthroplasty; *IR*, internal rotation; *ROD*, rate of decline; *ROM*, range of motion.

**Figure 4:**
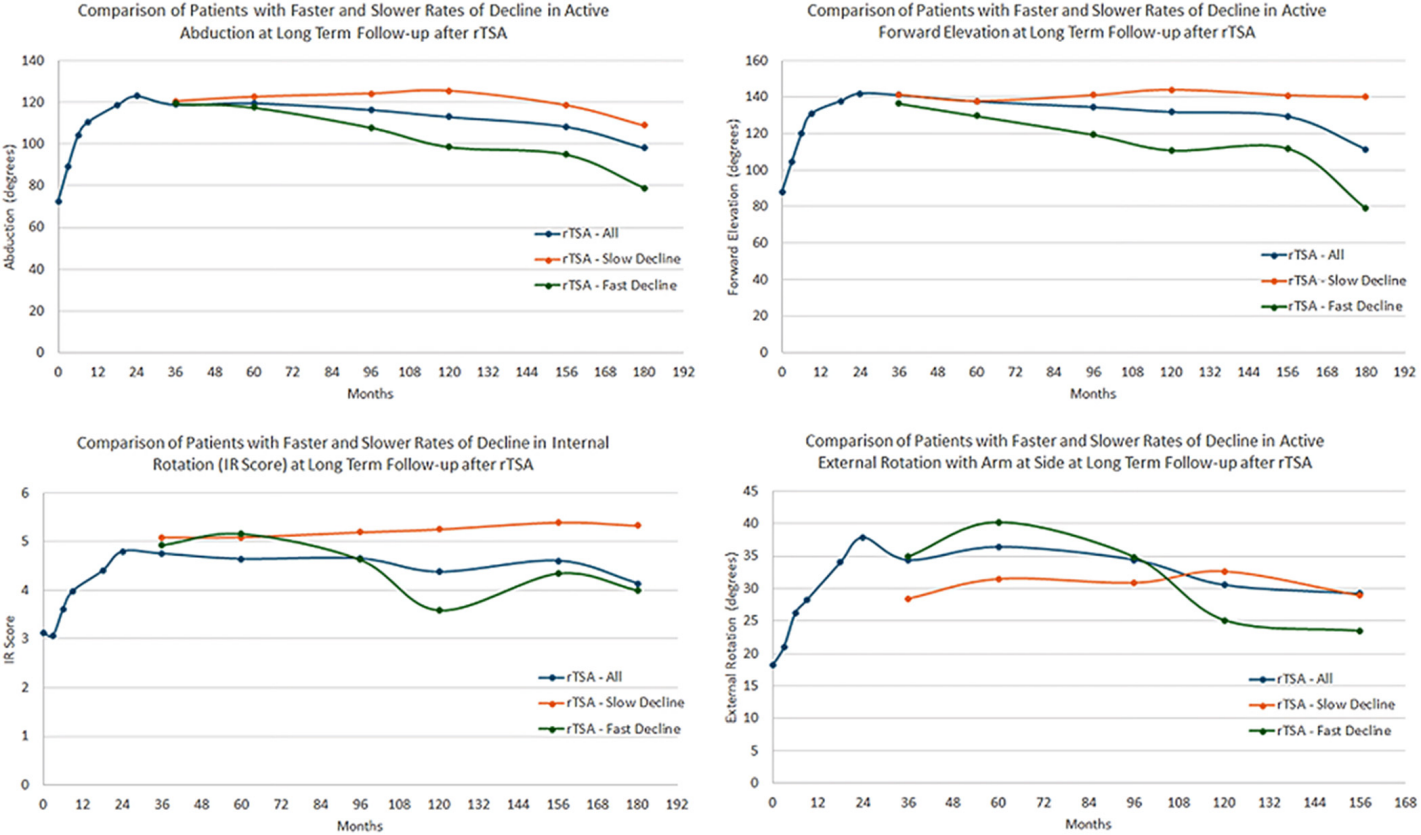
Comparison of active ROM in abduction (*top left*), forward elevation (*top right*), IR score (*bottom left*), and external rotation with arm at side (*bottom right*) for rTSA patients with a slow ROD (sustained outcomes) in ROM vs. fast ROD (not sustained outcomes) in ROM at long-term (>8 years) follow-up. *rTSA*, reverse total shoulder arthroplasty; *IR*, internal rotation; *ROD*, rate of decline; *ROM*, range of motion.

At the latest long-term follow-up, both aTSA and rTSA patients with a slow ROD in ROM had significantly better outcomes for every clinical measure as compared to aTSA (Table VI) and rTSA (Table VII) patients with a fast ROD. Supplementary Tables S1 and S2 demonstrate no differences in preoperative clinical outcomes between aTSA patients with a slow and fast ROD (Supplementary Table 1) and rTSA patients with a slow and fast ROD (Supplementary Table 2). As depicted in Figures 3 and 4, small declines in ROM were observed for each measure at long-term follow-up relative to peak ROM improvement for both aTSA (Fig. 3) and rTSA patients (Fig. 4), with the exception of the slow ROD rTSA cohort for active external rotation, which had a lower peak ROM than the other rTSA cohorts. Comparing Tables V and VI demonstrates that aTSA patients on average experience a decline from 2- to 3-year peak improvement to 8+-year long-term follow-up of 16° abduction, 12° forward elevation, 10° external rotation, and 1 IR score. Comparing Tables V and VII demonstrates that rTSA patients on average experience a decline from 2- to 3-year peak improvement to 8+-year long-term follow-up of 8° abduction, 10° forward elevation, 3° external rotation, and 0.5 IR score.

**Table VI:** Comparison of latest follow-up outcomes for primary aTSA patients with slow and fast ROD at long-term (>8 year) follow-up.

Follow-up duration	Active abduction (post/improve)	Active forward elevation (post/improve)	Active external rotation (post/improve)	IR score (post/improve)	VAS pain (post/improve)	Global shoulder function (post/improve)	ASES (post/improve)	Constant (post/improve)	Shoulder arthroplasty smart (post/improve)
Full aTSA cohort Latest follow-up of 118.1 mo	115.8 ± 33.6/30.4 ± 37.4	138.2 ± 33.3/37.6 ± 38.0	43.8 ± 19.4/27.2 ± 23.4	4.5 ± 1.6/1.6 ± 1.9	2.0 ± 2.7/4.6 ± 3.2	7.8 ± 2.3/3.9 ± 2.8	77.8 ± 23.5/43.5 ± 26.4	63.1 ± 16.8/24.1 ± 18.1	72.9 ± 15.6/27.8 ± 16.1
Slow ROD aTSA Latest follow-up of 137.9 mo	131.1 ± 25.4/47.0 ± 29.9	152.2 ± 21.6/52.0 ± 31.3	51.7 ± 19.4/36.9 ± 15.1	5.4 ± 1.2/2.1 ± 1.5	1.2 ± 2.0/5.0 ± 2.3	8.4 ± 1.9/4.0 ± 2.4	85.8 ± 17.9/48.3 ± 20.1	72.3 ± 11.4/31.8 ± 13.8	81.7 ± 11.7/33.3 ± 13.7
Fast ROD aTSA Latest follow-up of 130.0 mo	89.8 ± 29.9/7.3 ± 39.4	108.4 ± 36.5/37.6 ± 38.0	35.8 ± 18.7/27.2 ± 23.4	3.7 ± 1.5/1.6 ± 1.9	3.6 ± 3.1/4.6 ± 3.2	5.9 ± 2.4/3.9 ± 2.8	59.0 ± 25.2/43.5 ± 26.4	48.8 ± 17.0/24.1 ± 18.1	61.3 ± 16.2/27.8 ± 16.1
*P* value (slow ROD vs. fast ROD)	<.0001/<.0001	<.0001/<.0001	<.0001/.0002	<.0001/<.0001	<.0001/.0012	<.0001/.0005	<.0001/<.0001	<.0001/<.0001	<.0001/<.0001

*aTSA*, anatomic total shoulder arthroplasty; *ROD*, rate of decline; *IR*, internal rotation; *ASES*, American Shoulder and Elbow Surgeons; *VAS*, visual analog scale.

**Table VII:** Comparison of latest follow-up outcomes for primary rTSA patients with slow and fast ROD at long-term (>8 year) follow-up.

Follow-up duration	Active abduction (post/improve)	Active forward elevation (post/improve)	Active external rotation (post/improve)	IR score (post/improve)	VAS pain (post/improve)	Global shoulder function (post/improve)	ASES (post/improve)	Constant (post/improve)	Shoulder arthroplasty smart (post/improve)
Full rTSA cohort Latest follow-up of 108.4 mo	112.8 ± 29.2/36.4 ± 39.1	132.5 ± 28.3/39.6 ± 40.7	33.0 ± 18.8/14.6 ± 25.5	4.3 ± 1.8/1.0 ± 2.4	1.4 ± 2.3/4.6 ± 3.0	7.7 ± 2.1/4.0 ± 2.7	79.4 ± 20.1/42.9 ± 23.3	64.2 ± 16.4/27.2 ± 17.9	72.2 ± 14.0/24.8 ± 17.0
Slow ROD rTSA Latest follow-up of 122.2 mo	124.5 ± 26.0/41.1 ± 38.6	143.7 ± 19.5/49.0 ± 35.5	34.8 ± 21.3/17.6 ± 28.2	5.2 ± 1.5/1.8 ± 2.2	1.0 ± 1.7/5.0 ± 2.4	8.2 ± 1.9/5.0 ± 2.6	81.3 ± 15.1/45.4 ± 19.1	71.4 ± 9.8/36.6 ± 11.6	78.2 ± 9.8/33.7 ± 12.4
Fast ROD rTSA Latest follow-up of 124.8 mo	95.8 ± 26.0/18.2 ± 34.9	109.5 ± 27.6/19.4 ± 45.3	23.2 ± 20.1/-0.1 ± 28.5	3.5 ± 1.7/-0.3 ± 2.4	2.4 ± 3.4/3.8 ± 3.2	6.8 ± 2.6/2.9 ± 2.9	70.3 ± 28.0/33.1 ± 29.8	51.6 ± 19.2/15.6 ± 24.5	64.4 ± 16.6/14.7 ± 21.3
*P* value (slow ROD vs. fast ROD)	<.0001/.0077	<.0001/.0013	.0149/.0068	<.0001/.0001	.0094/.0759	.0039/.0015	.0183/.0322	<.0001/<.0001	<.0001/<.0001

*rTSA*, reverse total shoulder arthroplasty; *ROD*, rate of decline; *IR*, internal rotation; *ASES*, American Shoulder and Elbow Surgeons; *VAS*, visual analog scale.

A univariate and multivariate regression analysis identified patient (aTSA: Table VIII and rTSA: Table IX) and surgical/implant (aTSA: Supplementary Table S3 and rTSA: Supplementary Table S4) risk factors associated with a slow ROI. As described in Table VIII, aTSA patients with a slow ROI had significantly higher preoperative abduction (*P* = .020), IR score (<0.001), and external rotation (*P* < .001) as compared to aTSA patients with a fast/average ROI. As described in Table IX, rTSA patients with a slow ROI were more likely to have a diagnosis of diabetes (*P* = .049), were more likely to have previous injections (*P* = .022), and had significantly higher preoperative abduction (*P* < .001) and IR score (*P* = .001) as compared to rTSA patients with a fast/average ROI. Regarding implant differences (Supplementary Table S3), aTSA patients with a slow ROI were more likely to receive a large size (4.5 mm) replicator plate as compared to aTSA patients with a fast/average ROI. No implant differences were observed between rTSA patients with a slow and fast/average ROI (Supplementary Table S4).

**Table VIII:** Comparison of patient factors (demographics, diagnosis, comorbidities, preoperative ROM) associated with aTSA patients having a fast/average ROI of ROM improvement vs. aTSA patients having a slow ROI of ROM improvement from 0 to 2 years.

aTSA ROI - patient factors	Fast/average ROI	Slow ROI	*P* value (univariate)	*P* value (multivariate)	OR (95% CI) reference group = fast ROI
Age	65.1 ± 8.2	65.0 ± 8	.932		
Gender (% female)	48.3%	48.3%	1.000		
BMI	29.9 ± 6.3	30.3 ± 7.4	.515		
Previous surgery	11.8%	18.8%	**.036**	.179	1.5 (0.83-2.72)
Diagnosis					
Osteoarthritis	92.4%	94.5%	.472		
Osteonecrosis	2.5%	3.3%	.590		
Rotator cuff tear	3.9%	0.0%	**.004**	.190	0.22 (0-1.77)
Cuff tear arthropathy	0.6%	1.1%	.606		
Rheumatoid arthritis	3.4%	1.7%	.406		
Comorbidities					
None	36.2%	41.8%	.264		
Hypertension	48.4%	44.4%	.431		
Heart disease	11.9%	13.1%	.764		
Diabetes	11.2%	11.8%	.877		
Tobacco use	5.8%	7.2%	.546		
Chronic renal failure	0.3%	1.3%	.251		
Injections	40.1%	41.7%	.780		
Preoperative range of motion					
Abduction	75.5 ± 24.7	102.2 ± 29.7	**<.001**	**.020**	1.02 (1-1.03)
Forward elevation	89.8 ± 27.1	114.6 ± 30.3	**<.001**	.096	1.01 (1-1.02)
IR score	2.7 ± 1.5	3.9 ± 1.5	**<.001**	**<.001**	1.36 (1.18-1.58)
External rotation	12.9 ± 19	28.4 ± 18.2	**<.001**	**<.001**	1.03 (1.01-1.04)

*aTSA*, anatomic total shoulder arthroplasty; *ROI*, rate of improvement; *IR*, internal rotation; *ROM*, range of motion; *OR*, odds ratio; *CI*, confidence interval; *BMI*, body mass index.

Bold denotes *P* < .05.

**Table IX:** Comparison of patient factors (demographics, diagnosis, comorbidities, preoperative ROM) associated with rTSA patients having a fast/average ROI of ROM improvement vs. rTSA patients having a slow ROI of ROM improvement from 0 to 2 years

rTSA ROI - patient factors	Fast/average ROI	Slow ROI	*P* value (univariate)	*P* value (multivariate)	OR (95% CI) reference group = fast ROI
Age	71.0 ± 6.7	69.9 ± 7	.105		
Gender (% female)	67.0%	57.9%	**.049**	.609	0.87 (0.5-1.5)
BMI	28.9 ± 6.3	28.9 ± 6.7	.926		
Previous surgery	24.7%	37.8%	**.003**	.096	1.64 (0.91-2.95)
Diagnosis					
Osteoarthritis	55.2%	48.2%	.155		
Osteonecrosis	2.6%	1.2%	.516		
Rotator cuff tear	38.8%	45.1%	.179		
Cuff tear arthropathy	34.8%	48.8%	**.003**	.658	0.88 (0.49-1.54)
Rheumatoid arthritis	5.7%	4.3%	.534		
Comorbidities					
None	42.9%	42.4%	.920		
Hypertension	45.0%	49.3%	.424		
Heart disease	10.2%	15.3%	.123		
Diabetes	9.0%	17.4%	**.012**	**.049**	2.21 (1-4.87)
Tobacco use	5.7%	6.9%	.677		
Chronic renal failure	0.6%	0.7%	1.000		
Injections	30.3%	45.1%	**.001**	**.022**	1.88 (1.1-3.25)
Preoperative range of motion					
Abduction	59.4 ± 25.8	102.5 ± 32.5	**<.001**	**<.001**	1.04 (1.03-1.06)
Forward elevation	74.2 ± 32.1	117.0 ± 36.0	**<.001**	.183	1.01 (1-1.02)
IR score	2.6 ± 1.8	4.3 ± 1.8	**<.001**	**.001**	1.32 (1.13-1.55)
External rotation	14.4 ± 20	27.1 ± 21.5	**<.001**	.101	1.01 (1-1.03)

*rTSA*, reverse total shoulder arthroplasty; *ROI*, rate of improvement; *IR*, internal rotation; *ROM*, range of motion.

Bold denotes *P* < .05.

Similarly, a univariate and multivariate regression analysis identified patient (aTSA: Table X and rTSA: Table XI), surgical/implant (aTSA: Supplementary Table S5, rTSA: Supplementary Table S6), and other postoperative/radiographic risk factors (aTSA: Table XII, rTSA: Table XIII) associated with a fast ROD. As described in Table X, aTSA patients with a fast ROD were more likely to have heart disease (*P* = .002) as compared to aTSA patients with a slow/average ROD. As described in Table XI, rTSA patients with a fast ROD had significantly more comorbidities (*P* = .042) as compared to rTSA patients with a slow/average ROD. No implant differences were observed for aTSA (Supplementary Table S5) and rTSA (Supplementary Table S6) patients with a slow/average and fast ROD. Regarding postoperative/radiographic factors, as described in Table XII, aTSA patients with a fast ROD were more likely to have glenoid radiolucent lines (*P* = .005) than aTSA patients with a slow/average ROD. As described in Table XIII, rTSA patients with a fast ROD were more likely to experience a revision surgery (*P* = .011) than rTSA patients with a slow/average ROD.

**Table X:** Comparison of patient factors (demographics, diagnosis, comorbidities, preoperative ROM) associated with aTSA patients having a slow/average ROD in long-term ROM outcomes vs. aTSA patients having a fast ROD in long-term ROM outcomes.

aTSA ROD - patient factors	Slow ROD/average ROD	Fast ROD	*P* value (univariate)	*P* value (multivariate)	OR (95% CI) reference group = slow ROD
Age	64.6 ± 7.9	66.2 ± 7	.158		
Gender (% female)	53.8%	53.3%	1.000		
BMI	29.2 ± 6.2	31 ± 7.7	.101		
Previous surgery	14.5%	11.7%	.671		
Diagnosis					
Osteoarthritis	94.6%	93.3%	.751		
Osteonecrosis	2.7%	5.0%	.409		
Rotator cuff tear	2.2%	0.0%	.575		
Cuff tear arthropathy	1.1%	0.0%	1.000		
Rheumatoid arthritis	1.6%	3.3%	.599		
Comorbidities					
None	48.7%	33.3%	.088		
Hypertension	39.6%	51.1%	.175		
Heart disease	4.5%	20.0%	**.002**	**.002**	5.25 (1.84-15.61)
Diabetes	8.4%	17.8%	.096		
Tobacco use	5.8%	8.9%	.496		
Chronic renal failure	0.0%	2.2%	.226		
Injections	46.8%	40.7%	.455		

*aTSA*, anatomic total shoulder arthroplasty; *ROD*, rate of decline; *ROM*, range of motion.

Bold denotes *P* < .05.

**Table XI:** Comparison of patient factors (demographics, diagnosis, comorbidities, preoperative ROM) associated with rTSA patients having a slow/average ROD in long-term ROM outcomes vs. rTSA patients having a fast ROD in long-term ROM outcomes.

rTSA ROD - patient factors	Slow ROD/average ROD	Fast ROD	*P* value (univariate)	*P* value (multivariate)	OR (95% CI) reference group = slow ROD
Age	70.4 ± 6.7	69.6 ± 7.3	.557		
Gender (% female)	63.6%	54.5%	.333		
BMI	28.8 ± 6.7	29.4 ± 6.5	.658		
Previous surgery	32.7%	24.2%	.413		
Diagnosis					
Osteoarthritis	56.8%	42.4%	.179		
Osteonecrosis	3.1%	0.0%	.591		
Rotator cuff tear	36.4%	45.5%	.333		
Cuff tear arthropathy	43.8%	45.5%	1.000		
Rheumatoid arthritis	3.1%	9.1%	.136		
Comorbidities					
None	52.1%	31.0%	**.043**	**.042**	0.41 (0.17-0.95)
Hypertension	39.6%	48.3%	.413		
Heart disease	9.0%	17.2%	.190		
Diabetes	12.5%	6.9%	.534		
Tobacco use	3.5%	10.3%	.132		
Chronic renal failure	0.7%	0.0%	1.000		
Injections	39.1%	42.4%	.845		

*rTSA*, reverse total shoulder arthroplasty; *ROI*, rate of improvement; *ROD*, rate of decline; *ROM*, range of motion.

Bold denotes *P* < .05.

**Table XII:** Comparison of radiographical factors and complications associated with aTSA patients having a slow/average ROD in long-term ROM outcomes vs. aTSA patients having a fast ROD in long-term ROM outcomes.

aTSA ROD – radiographical factors and complications	Slow ROD/average ROD	Fast ROD	*P* value (univariate)	*P* value (multivariate)	OR (95% CI) reference group = slow ROD
Humeral radiolucent lines	18.6%	33.9%	**.025**	.569	1.25 (0.57-2.69)
Glenoid radiolucent lines	36.6%	77.8%	**<.001**	**.005**	4.6 (1.58-13.5)
Glenoid radiolucent line grade	1.0 ± 1.7	2.4 ± 1.9	**<.001**	.712	1.06 (0.79-1.41)
Complication	4.8%	15.0%	**.018**	.318	1.79 (0.56-5.6)
Revision	4.3%	11.7%	.058		

*aTSA*, anatomic total shoulder arthroplasty; *ROD*, rate of decline; *ROM*, range of motion.

Bold denotes *P* < .05.

**Table XIII:** Comparison of radiographical factors and complications associated with rTSA patients having a slow/average ROD in long-term ROM outcomes vs. rTSA patients having a fast ROD in long-term ROM outcomes.

rTSA ROD – radiographical factors and complications	Slow ROD/average ROD	Fast ROD	*P* value (univariate)	*P* value (multivariate)	OR (95% CI) reference group = slow ROD
Humeral radiolucent lines	30.8%	45.2%	.143		
Scapular notching	14.7%	12.9%	1.000		
Scapular notching grade	0.3 ± 0.9	0.2 ± 0.6	.353		
Complication	0.6%	6.1%	.075		
Revision	0.0%	6.1%	**.028**	**.011**	25.79 (2.04-3591.25)

*rTSA*, reverse total shoulder arthroplasty; *ROD*, rate of decline; *ROM*, range of motion.

Bold denotes *P* < .05.

## Discussion

The results of this 1272 patient long-term clinical outcome study demonstrates that aTSA and rTSA patients can experience different rates of ROM improvement and different rates of ROM decline. Differences in ROM improvement between these slow/fast ROI cohorts were generally isolated to the first 3 months after surgery, as patients with high preoperative ROM generally experienced a decline in ROM during those first 3 months, whereas patients with low preoperative ROM experienced substantial improvements in ROM during the first 3 months. Despite these initial differences, patients with fast, average, and slow ROI generally experienced the same ROI for each ROM measure between 3 and 24 months.

aTSA patients with a slow ROI had significantly higher preoperative abduction, IR score, and external rotation, whereas rTSA patients with a slow ROI were significantly more likely to have diabetes, injections, and significantly more preoperative abduction and IR score. aTSA patients with a fast ROD were significantly more likely to have heart disease and significantly more likely to have glenoid radiolucent lines. rTSA patients with a fast ROD were significantly more likely to have comorbidities and significantly more likely to experience a revision surgery. These findings are useful for postoperative patient counseling, as some slow ROI patients (particularly those who had considerable preoperative ROM) may become discouraged with a loss of ROM during the early recovery period; however, our findings suggest that these patients will, on average achieve clinical improvement by 1 year, ultimately achieving peak ROM that is comparable to the fast/average ROI cohort.

The multivariate analysis did not identify age or gender as significant factors influencing the short-term ROI or the long-term ROD. These findings are different than what was identified by Friedman et al, who conducted a linear mixed effects statistical analysis on 660 rTSA patients with 2 years of minimum follow-up to investigate the patient factors associated with ROM.^9^ When controlling for gender, they found each 1-year increase in age at the time of rTSA procedure was associated with a mean decrease in active abduction by 0.26° and a mean decrease of forward flexion by 0.39°.^9^ Age at the time of the rTSA procedure had no impact on external rotation. When controlling for age, they found that male patients achieved significantly more abduction (5.79°) and forward flexion (7.68°) as compared to female patients.^9^ In contrast, our multivariate analysis on 8-year minimum results identified that rTSA patients with a fast ROD in ROM were significantly more likely to have comorbidities and revision surgery, as compared to rTSA patients with an average/slow ROD in ROM.

The ROM reported for aTSA and rTSA at long-term follow-up was similar to that reported by numerous other studies.^1^^,^^2^^,^^4^^,^^8^^,^^10^^,^^11^^,^^19^^,^^25^^,^^35^^,^^36^ Additionally, several studies have previously reported differences in ROM improvement with aTSA and rTSA.^5^^,^^22^^,^^25^^,^^40^ Simovitch et al analyzed the rates and magnitudes of improvement of 505 aTSA and 678 rTSA with 2-years minimum follow-up and reported that the patterns of improvement between aTSA and rTSA were similar, with most improvement achieved by 6 months after surgery; however, some aTSA and rTSA patients continued to improve up to 24 months.^40^ Regarding differences in ROM improvement between aTSA and rTSA patients, Simovitch et al^40^ reported that rTSA patients experiencing significantly more improvement in forward elevation from 12 to 36 months but significantly less improvement from 72 to 84 months, as compared to aTSA patients. Conversely, aTSA patients experienced significantly more improvement in active external rotation from <3 to 84 months, as compared to rTSA patients.^40^ Similar patterns of improvement were identified in our study of 688 aTSA and 584 rTSA patients with 8-year minimum follow-up, with peak improvement achieved by 2 years for aTSA and rTSA patients and some gradual declines in ROM with follow-up duration. We also observed that aTSA patients experienced significantly more ROM than rTSA patients for all measures at latest follow-up. Regarding differences in ROD between aTSA and rTSA, Schoch et al investigated the phenomenon of “deltoid fatigue” by analyzing the ROD of overhead motion for 384 aTSA with 10-year minimum follow-up and 165 rTSA patients with minimum 5-year follow-up.^35^^,^^36^ Schoch et al reported that irrespective of patient age, gender, or preoperative diagnosis, aTSA and rTSA patients experienced a statistically equivalent loss of motion with follow-up duration, where specifically, aTSA patients lose 0.7°/year of abduction and rTSA patients lose 0.8°/year of forward elevation and abduction.^35^^,^^36^ In comparison, our study found that rTSA patients experienced less decline in ROM than aTSA patients from 2-3 year peak improvement to 8+ year long-term follow-up. Despite this difference, aTSA patients generally maintained greater ROM than rTSA patients at long-term follow-up.

The radiographic findings merit discussion. It is not surprising that a loss of aTSA glenoid implant–bone fixation is associated with a faster ROD of long-term aTSA outcomes. The presence of radiolucent glenoid lines has long been reported to be a harbinger of clinical loosening^7^^,^^16^^,^^19^^,^^23^ and some^8^^,^^36^ have reported that aTSA glenoids with higher radiolucent line grades are associated with worse clinical outcomes. Improvements in implant design, fixation, and alternative bearing surfaces may offer potential for longer sustained outcomes. Conversely, the finding that scapular notching was not a risk factor for a faster ROD of long-term rTSA outcomes was surprising, as many^11^^,^^24^^,^^34^^,^^39^ have demonstrated that scapular notching negatively impacts clinical outcomes. However, our results should be interpreted in context of the overall scapular notching rate at long-term follow-up, since the scapular notching rate in each cohort was low (high ROD = 12.9%; low/average ROD = 14.7%), and only 7 patients experienced a scapular notching grade >2.

This study has several limitations. First, this retrospective database analysis utilized data from multiple surgeons at 17 different clinical sites in the US and Europe, which introduces substantial variability in surgical technique and implant selection. Second, we only investigated ROM and clinical outcomes in patients with >8 years follow-up, excluding patients who did not have long-term follow-up and those lost to follow-up, as such, we acknowledge some selection bias may have occurred using this inclusion criteria. We also acknowledge that some may consider long-term follow-up to be longer than 8 years. Third, the method to classify patients into slow, average, and fast ROI and ROD cohorts was somewhat arbitrary and it may be that this selection criteria inadvertently influenced our findings. Relatedly, not all patients had enough follow-up visits to establish a trend and be classified into a slow/fast ROI/ROD. Fourth, no information related to postoperative rehabilitation was analyzed, as such, we were unable to quantify the impact of rehabilitation on the ROI. It may be that some rehabilitation methods improved the ROI more than others, irrespective of the amount of preoperative ROM. Fifth, radiographic evaluation was performed by the implanting surgeon and we did not utilize multiple reviewers to assess interobserver reliability. Sixth, the use of a logistic multivariate regression to identify risk factors has certain inherent limitations, while we analyzed numerous parameters, we did not collect/analyze all potentially relevant data. For example, we did not assess the impact of preoperative glenoid retroversion, beta angle, or the Walch and Favard classification, nor did we assess the impact of the soft tissue, like rotator cuff integrity, deltoid shape/size/volume, etc. Previous work has demonstrated that each of these parameters are associated with differences in clinical outcomes, complications, and revisions after aTSA and rTSA.^12^^,^^26^^,^^28^^,^^29^^,^^34^^,^^41^ Therefore, our analysis likely failed to consider all possible/relevant risk factors associated with ROI and ROD.

## Conclusion

This aTSA/rTSA clinical outcome study quantified and compared the ROI in ROM during the 2-year recovery period and the ROD in ROM at long-term follow-up. Several risk factors were identified for a slow ROI and a fast ROD. Most notably, a patient's ROI in ROM was highly dependent on their preoperative ROM. Where generally, patients with low preoperative ROM experienced a faster ROI than patients with high preoperative ROM. Additionally, we found that patients with high preoperative ROM experienced, on average, declines in ROM during the first 3 months, but later recovered at a similar rate and achieved similar peak improvements as patients with a faster ROI. Patients with a fast ROD at long-term follow-up were generally impacted by systemic health issues (ie, heart disease and more comorbidities), compromised implant fixation (ie, radiolucent lines after aTSA), and the onset of revision surgery. These findings may be beneficial for patient counseling, to manage expectations and help patients better understand when to expect ROM improvement after aTSA and rTSA, depending on their amount of preoperative ROM, and especially to encourage patients who may have experienced a decline (or little improvement) in ROM during the first 3 months after surgery.

## Disclaimers

Funding: No funding was provided to complete this particular study. Funding was provided by Exactech Inc for the multicenter clinical data collection program, which provided the data used in this study.

Conflicts of interest: Christopher Roche and Josie Elwell are employed by Exactech, Inc. Richard Jones, Howard Routman, Ryan Simovitch, Pierre-Henri Flurin, Thomas Wright, and Joseph Zuckerman are consultants for Exactech and receive royalties on products discussed in this paper.
