# Supplementary material for: Analysis of patients with differing short-term rates of improvement and long-term rates of decline in range of motion and after anatomic and reverse total shoulder arthroplasty
Source: JSES Int. 2025 May 14;9(4):1327–38. doi: 10.1016/j.jseint.2025.04.018 (PMC12435041; doi:10.1016/j.jseint.2025.04.018)
Supplement: Supplementary Table S1 [file mmc1.docx]

**Supplemental Table 1**. Comparison of Preoperative Outcomes for the Primary aTSA Patients with Slow ROD and Fast ROD at Long-Term (>8 years) Follow-up

| aTSA Preoperative Comparison | Active Abduction | Active Forward Elevation | Active External Rotation | IR Score | VAS Pain | Global Shoulder Function | ASES | Constant | Shoulder Arthroplasty Smart |
| --- | --- | --- | --- | --- | --- | --- | --- | --- | --- |
| Full aTSA Cohort Preoperative | 84.8 ± 29.7 | 99.2 ± 31.5 | 17.6 ± 20.1 | 3.1 ± 1.6 | 6.4 ± 2.0 | 4.0 ± 2.0 | 35.5 ± 15.6 | 38.0 ± 13.4 | 45.9 ± 10.4 |
| Slow ROD: Sustained Long-term aTSA Improvement  Preoperative | 83.5 ± 27.2 | 99.3 ± 30.5 | 15.1 ± 20.0 | 3.3 ± 1.5 | 6.1 ± 1.5 | 4.3 ± 1.5 | 38.2 ± 12.8 | 40.3 ± 11.2 | 47.3 ± 9.5 |
| Fast ROD: Not Sustained Long-term aTSA Improvement  Preoperative | 82.1 ± 28.4 | 96.2 ± 30.5 | 14.8 ± 20.1 | 2.9 ± 1.6 | 6.1 ± 2.1 | 4.0 ± 1.7 | 36.9 ± 13.8 | 37.5 ± 13.9 | 45.1 ± 10.4 |
| P-Value (Slow ROD vs. Fast ROD) | 0.7710 | 0.5810 | 0.9277 | 0.1268 | 0.9646 | 0.4313 | 0.6319 | 0.2872 | 0.2663 |
