# Supplementary material for: Analysis of patients with differing short-term rates of improvement and long-term rates of decline in range of motion and after anatomic and reverse total shoulder arthroplasty
Source: JSES Int. 2025 May 14;9(4):1327–38. doi: 10.1016/j.jseint.2025.04.018 (PMC12435041; doi:10.1016/j.jseint.2025.04.018)
Supplement: Supplementary Table S2 [file mmc2.docx]

**Supplemental Table 2**. Comparison of Preoperative Outcomes for the Primary rTSA Patients with Slow ROD and Fast ROD at Long-Term (>8 years) Follow-up

| rTSA Preoperative Comparison | Active Abduction | Active Forward Elevation | Active External Rotation | IR Score | VAS Pain | Global Shoulder Function | ASES | Constant | Shoulder Arthroplasty Smart |
| --- | --- | --- | --- | --- | --- | --- | --- | --- | --- |
| Full rTSA Cohort Preoperative | 72.6 ± 34.5 | 88.0 ± 38.9 | 18.2 ± 21.6 | 3.1 ± 1.9 | 6.0 ± 2.3 | 3.7 ± 2.0 | 36.3 ± 16.1 | 34.6 ± 14.0 | 45.5 ± 12.0 |
| Slow ROD: Sustained Long-term rTSA Improvement  Preoperative | 82.2 ± 35.1 | 93.8 ± 41.2 | 17.1 ± 24.0 | 3.4 ± 1.9 | 5.8 ± 2.1 | 3.4 ± 1.8 | 38.3 ± 13.9 | 36.0 ± 12.4 | 45.9 ± 10.6 |
| Fast ROD: Not Sustained Long-term rTSA Improvement  Preoperative | 76.3 ± 35.4 | 88.6 ± 45.3 | 23.9 ± 25.5 | 3.5 ± 1.8 | 6.3 ± 2.4 | 3.9 ± 2.0 | 35.7 ± 18.2 | 35.7 ± 17.7 | 47.0 ± 15.2 |
| P-Value (Slow ROD vs. Fast ROD) | 0.4554 | 0.5841 | 0.2097 | 0.8230 | 0.4156 | 0.2208 | 0.4784 | 0.9306 | 0.7332 |
