# Supplementary material for: Analysis of patients with differing short-term rates of improvement and long-term rates of decline in range of motion and after anatomic and reverse total shoulder arthroplasty
Source: JSES Int. 2025 May 14;9(4):1327–38. doi: 10.1016/j.jseint.2025.04.018 (PMC12435041; doi:10.1016/j.jseint.2025.04.018)
Supplement: Supplementary Table S3 [file mmc3.docx]

**Supplemental Table 3**. Comparison of Surgical Factors (Implant Type, Implant Size) Associated with aTSA Patients having a Fast/Average ROI of ROM improvement vs. aTSA Patients having a Slow ROI of ROM improvement from 0 to 2 years

| **aTSA ROI – Surgical Factors** | **Fast/Average  ROI** | **Slow ROI** | **p  (univariate)** | **p (multivariate)** | **OR (95% CI) Reference group = Fast ROI** |
| --- | --- | --- | --- | --- | --- |
| Cemented Stem | 22.1% | 21.5% | 0.913 |  |  |
| Humeral Head Thickness (% extra short/short) | 83.1% | 80.7% | 0.546 |  |  |
| Humeral Head Diameter | 45.9 ± 3.5 | 46 ± 3.5 | 0.800 |  |  |
| Radial Mismatch | 5.4 ± 1.1 | 5.4 ± 1.1 | 0.694 |  |  |
| Replicator Plate Offset (% 4.5mm) | 68.5% | 79.4% | **0.046** | **0.044** | 1.77 (1.03, 3.16) |
| Glenoid Type  (% hybrid cage) | 35.9% | 43.1% | 0.111 |  |  |
| Augmented Glenoid | 10.4% | 8.3% | 0.537 |  |  |
| Glenoid Size  (% small/medium) | 63.6% | 62.7% | 0.892 |  |  |
