# Supplementary material for: Analysis of patients with differing short-term rates of improvement and long-term rates of decline in range of motion and after anatomic and reverse total shoulder arthroplasty
Source: JSES Int. 2025 May 14;9(4):1327–38. doi: 10.1016/j.jseint.2025.04.018 (PMC12435041; doi:10.1016/j.jseint.2025.04.018)
Supplement: Supplementary Table S4 [file mmc4.docx]

**Supplemental Table 4**. Comparison of Surgical Factors (Implant Type, Implant Size) Associated with rTSA Patients having a Fast/Average ROI of ROM improvement vs. rTSA Patients having a Slow ROI of ROM improvement from 0 to 2 years

| **rTSA ROI – Surgical Factors** | **Fast/Average  ROI** | **Slow ROI** | **p  (univariate)** | **p (multivariate)** | **OR (95% CI) Reference group = Fast ROI** |
| --- | --- | --- | --- | --- | --- |
| Subscapularis Repair | 48.1% | 40.7% | 0.162 |  |  |
| Cemented Stem | 8.0% | 9.7% | 0.613 |  |  |
| Glenosphere Diameter > 38mm | 67.5% | 62.6% | 0.438 |  |  |
| Constrained Humeral Liner | 1.0% | 3.0% | 0.337 |  |  |
| Liner/tray Offset > 0mm | 74.7% | 79.5% | 0.448 |  |  |
| Expanded Glenosphere | 6.2% | 5.7% | 1.000 |  |  |
| Augmented Baseplate | 17.9% | 17.9% | 1.000 |  |  |
