# Supplementary material for: Analysis of patients with differing short-term rates of improvement and long-term rates of decline in range of motion and after anatomic and reverse total shoulder arthroplasty
Source: JSES Int. 2025 May 14;9(4):1327–38. doi: 10.1016/j.jseint.2025.04.018 (PMC12435041; doi:10.1016/j.jseint.2025.04.018)
Supplement: Supplementary Table S5 [file mmc5.docx]

**Supplemental Table 5**. Comparison of Surgical Factors (Implant Type, Implant Size) Associated with aTSA Patients having a Slow/Average ROD in Long-Term ROM Outcomes vs. aTSA Patients having a Fast ROD in Long-Term ROM Outcomes

| **aTSA ROD – Surgical Factors** | **Slow ROD/**  **Average ROD** | **Fast ROD** | **p  (univariate)** | **p (multivariate)** | **OR (95% CI) Reference group = Slow ROD** |
| --- | --- | --- | --- | --- | --- |
| Cemented Stem | 16.2% | 11.7% | 0.534 |  |  |
| Humeral Head Thickness (% extra short/short) | 85.2% | 86.2% | 1.000 |  |  |
| Humeral Head Diameter | 45.9 ± 3 | 46 ± 3.6 | 0.910 |  |  |
| Radial Mismatch | 5.3 ± 1.1 | 5.2 ± 1 | 0.457 |  |  |
| Replicator Plate Offset (% 4.5mm) | 72.3% | 70.6% | 0.828 |  |  |
| Glenoid Type  (% hybrid cage) | 29.0% | 18.3% | 0.129 |  |  |
| Augmented Glenoid | 9.1% | 6.7% | 0.791 |  |  |
| Glenoid Size  (% small/medium) | 64.5% | 60.0% | 0.663 |  |  |
