# Supplementary material for: Analysis of patients with differing short-term rates of improvement and long-term rates of decline in range of motion and after anatomic and reverse total shoulder arthroplasty
Source: JSES Int. 2025 May 14;9(4):1327–38. doi: 10.1016/j.jseint.2025.04.018 (PMC12435041; doi:10.1016/j.jseint.2025.04.018)
Supplement: Supplementary Table S6 [file mmc6.docx]

**Supplemental Table 6**. Comparison of Surgical Factors (Implant Type, Implant Size) Associated with rTSA Patients having a Slow/Average ROD in Long-Term ROM Outcomes vs. rTSA Patients having a Fast ROD in Long-Term ROM Outcomes

| **rTSA ROD – Surgical Factors** | **Slow ROD/**  **Average ROD** | **Fast ROD** | **p  (univariate)** | **p (multivariate)** | **OR (95% CI) Reference group = Slow ROD** |
| --- | --- | --- | --- | --- | --- |
| Subscapularis Repair | 47.1% | 51.7% | 0.689 |  |  |
| Cemented Stem | 6.2% | 6.1% | 1.000 |  |  |
| Glenosphere Diameter > 38mm | 65.5% | 64.3% | 1.000 |  |  |
| Constrained Humeral Liner | 0.0% | 0.0% | - |  |  |
| Liner/tray Offset > 0mm | 82.3% | 91.7% | 0.682 |  |  |
| Expanded Glenosphere | 9.2% | 8.3% | 1.000 |  |  |
| Augmented Baseplate | 17.8% | 17.2% | 1.000 |  |  |
